# Behavioral Characterization of β-Arrestin 1 Knockout Mice in Anxiety-Like and Alcohol Behaviors

**DOI:** 10.3389/fnbeh.2018.00054

**Published:** 2018-03-20

**Authors:** Meridith T. Robins, Terrance Chiang, Jennifer N. Berry, Mee Jung Ko, Jiwon E. Ha, Richard M. van Rijn

**Affiliations:** Department of Medicinal Chemistry and Molecular Pharmacology, Purdue Institute for Integrative Neuroscience, Purdue University, West Lafayette, IN, United States

**Keywords:** β-arrestin 1, alcohol, anxiety, knockout mice, sex difference

## Abstract

β-Arrestin 1 and 2 are highly expressed proteins involved in the desensitization of G protein-coupled receptor signaling which also regulate a variety of intracellular signaling pathways. Gene knockout (KO) studies suggest that the two isoforms are not homologous in their effects on baseline and drug-induced behavior; yet, the role of β-arrestin 1 in the central nervous system has been less investigated compared to β-arrestin 2. Here, we investigate how global β-arrestin 1 KO affects anxiety-like and alcohol-related behaviors in male and female C57BL/6 mice. We observed increased baseline locomotor activity in β-arrestin 1 KO animals compared with wild-type (WT) or heterozygous (HET) mice with a sex effect. KO male mice were less anxious in a light/dark transition test, although this effect may have been confounded by increased locomotor activity. No differences in sucrose intake were observed between genotypes or sexes. Female β-arrestin 1 KO mice consumed more 10% alcohol than HET females in a limited 4-h access, two-bottle choice, drinking-in-the-dark model. In a 20% alcohol binge-like access model, female KO animals consumed significantly more alcohol than HET and WT females. A significant sex effect was observed in both alcohol consumption models, with female mice consuming greater amounts of alcohol than males relative to body weight. Increased sensitivity to latency to loss of righting reflex (LORR) was observed in β-arrestin 1 KO mice although no differences were observed in duration of LORR. Overall, our efforts suggest that β-arrestin 1 may be protective against increased alcohol consumption in females and hyperactivity in both sexes.

## Introduction

Arrestins are a family of auxiliary proteins associated with GPCRs that consist of four members: visual arrestin (arrestin 1), cone arrestin (arrestin 4), β-arrestin 1 (arrestin 2), and β-arrestin 2 (arrestin 3) (Patwari and Lee, 2012). The non-visual β-arrestin subtypes 1 and 2 are both highly expressed in the central nervous system (CNS) (Bychkov et al., 2012) and are heavily implicated in negative regulation of GPCR signaling; β-arrestins can uncouple the G-protein from receptors to “arrest” signaling and assist in receptor internalization (Lohse et al., 1990; Oakley et al., 2000, 2001; Laporte et al., 2002; Tian et al., 2014), but interestingly have also been found capable of initiating signal transduction at GPCRs in a G-protein-independent manner (Ferguson et al., 1996; McDonald et al., 2000; Laporte et al., 2002; DeWire et al., 2007).

The critical importance of β-arrestins on neurophysiology was first demonstrated through studies using β-arrestin 2 KO animals in which mice lacking β-arrestin 2 failed to develop antinociceptive tolerance and sensitization to chronic morphine, thereby implicating β-arrestin 2 in opioid tolerance (Bohn et al., 1999, 2000). Since the first reports of differing drug responses between WT and β-arrestin 2 KO animals in 1999, both pharmaceutical companies and academic laboratories have been eager to develop molecules that either preferably recruit and signal via β-arrestins over G proteins (Allen et al., 2011; Mannel et al., 2017b; Teixeira et al., 2017) or, conversely, that are G-protein-biased (Soergel et al., 2013, 2014; Chiang et al., 2016; Manglik et al., 2016; Singla et al., 2016) in order to treat neurophysiological disorders while avoiding adverse side effects. In these endeavors, however, little attention is given to the different β-arrestin isoforms, β-arrestin 1 and 2. While β-arrestin 1 or β-arrestin 2 global KO mice are viable and healthy, each display altered physiology and behavior compared with WT mice (Bjork et al., 2008; Zurkovsky et al., 2017). In contrast, double KO mice are embryonically lethal, indicating a degree of functional compensation and overlap within the isoforms (Ikeda et al., 2007). Still, despite the high 78% sequence homology between the β-arrestin 1 and 2 (Attramadal et al., 1992), differential expression and functionality of the two isoforms has been observed in various CNS disorders (Wang et al., 2014) and following drug exposure (Fan et al., 2003).

In general, compared to β-arrestin 2 KO mice, fewer studies have investigated behavioral responses in β-arrestin 1 KO mice, although the role of β-arrestin 1 has been investigated in non-CNS contexts such as cancer (Wang et al., 2012; Gatto et al., 2013), autoimmunity (Shi et al., 2007), and cardiology (Abraham et al., 2016). Recently, a study by Zurkovsky et al. (2017) found that β-arrestin 2 KO mice exhibit reduced amphetamine-induced locomotion, while β-arrestin 1 KO mice exhibit increased locomotor responsiveness to amphetamine. This divergent modulation of amphetamine-induced locomotion further suggests that the β-arrestin isoforms may differentially contribute to drug-related behaviors. Additionally, most studies that have used β-arrestin KO mice in the past have solely used male mice; therefore, we decided to characterize both male and female WT, HET, and homozygous β-arrestin 1 KO C57BL/6 mice in animal models of reward intake and anxiety-like behavior. Here, we observed β-arrestin 1-specific changes (with sex-specific effects) in behaviors, such as increased locomotor activity, increased alcohol intake, and faster alcohol-induced sedation.

## Materials and Methods

### Drugs and Chemicals

Two hundred proof ethyl alcohol and sucrose were purchased from Sigma-Aldrich (St. Louis, MO, United States).

### Animal Husbandry

β-Arrestin 1 KO C57BL/6 mice were obtained from the laboratory of Dr. Amynah Pradhan at the University of Illinois–Chicago, but originated in the laboratory of Dr. Robert Lefkowitz (Duke University) (Kohout et al., 2001; Pradhan et al., 2016). The original KO strain was created by crossing a 129/Sv strain with a black Swiss strain (Conner et al., 1997), but was then backcrossed to C57BL/6 mice for more than 10 generations. The β-arrestin 1 (-/-) KO C57BL/6 mice were obtained via β-arrestin 1 (-/-) × β-arrestin 1 (-/-) breeding pairs. The β-arrestin 1 (+/-) HET KO were obtained via β-arrestin 1 (-/-) × WT C57BL/6 mice breeding pairs.

Food was provided *ad libitum*; water was provided *ad libitum* unless specified for ethyl alcohol and sucrose consumption experiments. Throughout the experiment, animals were housed in Plexiglas cages in ventilated racks at ambient temperature of (21°C) in a room maintained on a reversed 12L:12D cycle (lights off at 10.00, lights on at 22.00) in Purdue University’s animal facility, which is accredited by the Association for Assessment and Accreditation of Laboratory Animal Care. This study was carried out in accordance with the recommendations of the National Institutes of Health Guide for the Care and Use of Laboratory Animals. The protocol was approved by the Purdue University Institutional Animal Care and Use Committee (No. 1305000864).

In designing the behavior assays, two separate experimental batteries were created to efficiently run the experiments while reducing/refining the number of mice necessary. All behavioral experiments were performed during the dark/active phase of the mice. Mice in the first group were tested for locomotor activity/open-field (2 days total) 3 days prior to alcohol intake studies, which took 5 weeks total (3 weeks 10%, followed by 2 weeks 20%). Mice assigned to the second group underwent light/dark transition testing (1 day) 3 days prior to sucrose intake assays (10 days total). Five days later, LORR (1 day) was assessed in this second group. All mice were approximately 55 days post-natal at the beginning of testing.

### Western Blot Analysis of β-Arrestin 1 Expression

Mice were euthanized for rapid brain dissection by CO_2_ asphyxiation and brain samples were stored on dry ice following organ harvest. For sample preparation, 30 mg of cerebellar tissue was lysed using 200 μL RIPA buffer and 1× protease inhibitors (ThermoFisher, Waltham, MA, United States). Samples were then homogenized using a manual hand-homogenizer and sonicated prior to centrifugation at 12,000 rpm for 20 min at 4°C, where the supernatant was kept for further analysis and pellet was discarded. Protein concentration was determined by Bradford protein determination assay (Bio-Rad, Hercules, CA, United States) and 10 mg protein/20 μL sample was loaded into a gradient gel (Life Technologies, Carlsbad, CA, United States). Following gel transfer to a nitrocellulose membrane (ThermoFisher, Waltham, MA, United States) and blocking (Li-Cor Blocking Buffer, Lincoln, NE, United States), the membrane was stained using mouse anti-α-tubulin (SC-5286, Lot No. 63117, Santa Cruz Biotechnology, Dallas, TX, United States) and rabbit anti-β-arrestin 1 XP^®^ (D723W, 300365, Lot No. 1, Cell Signaling, Danvers, MA, United States) primary antibodies, both at a 1:1000 dilution in blocking buffer with 0.2% Tween for 1 h at room temperature. Secondary antibodies IRdye 680 LT goat anti-mouse (925-68020, Lot No. C60824-02 Li-Cor Blocking Buffer, Lincoln, NE, United States) and IRdye 800 CW goat anti-rabbit (925-32211, Lot No. C61103-06, Li-Cor Blocking Buffer, Lincoln, NE, United States) were applied 1:5000 in blocking buffer for 1 h at room temperature. The membrane was then imaged using Li-Cor Odyssey CLx and relative β-arrestin 1 protein concentration was determined by normalized intensity to loading α-tubulin (loading control) as well as average normalized β-arrestin 1 protein concentration of WT mice (genotype control) using ImageJ software (NIH, Bethesda, MA, United States).

### Open-Field Locomotion

Square locomotor boxes from Med Associates (L 27.3 cm × W 27.3 cm × H 20.3 cm, St. Albans, VT, United States) were used to monitor locomotor activity. For all locomotor studies, animals were moved to the testing room for 60 min prior to testing. A 60-min habituation session to the boxes was conducted 1 day prior to testing, to avoid measuring locomotor activity driven by novelty and/or anxiety. During this habituation session for the open-field test, anxiety-like behavior was measured. For open-field anxiety testing, the time spent in the center of the area (12 × 12 cm) during the first 5 min of the testing session and the total number of crosses into the center area were recorded. The following day, locomotor activity was monitored for a total of 60 min for analysis. All testing was conducted during the dark/active light phase.

### Light/Dark Box

Animals were habituated to the experimental room >30 min prior to testing. Testing was conducted without a habituation session to the boxes and a one-third area dark insert was placed in the locomotor boxes, leaving the remaining two-third of the area lit as described previously (Bourin and Hascoet, 2003). Two LED lights were inserted above the light portion of the testing chamber where the lux of the light region ranged from 390 to 540 lumens and dark chamber lux ranged from 0 to 12 lumens. For testing, animals were placed in the light portion of the chamber and testing began upon animal entry. Analysis of the 10 min sessions utilized MedAssociates software to measure total locomotion per area, total time spent in area, total crosses between areas, and latency to enter the dark area (Crawley and Goodwin, 1980; Bourin and Hascoet, 2003; Takao and Miyakawa, 2006). All testing was conducted during the dark/active light phase.

### Sucrose Consumption

Mice were individually housed in double-grommet ventilated Plexiglas cages to monitor individual fluid consumption. Animals were offered increasing concentrations of sucrose in water (0.25–4%) in 2-day increments for a total of 10 days where the total amount of sucrose liquid was recorded daily (Bachmanov et al., 2001; Robins et al., 2016). Sucrose was offered in a two-bottle (sucrose vs. reverse osmosis water), limited-access (4 h) choice 2 h into the dark/active phase, where the location of each bottle was alternated daily (right vs. left grommet) to prevent habit formation. After each drinking session, bottles were removed and weighed for total volume water and sucrose consumed during the 4-h session. Water intake during the 20 h single access to water was measured for changes in general fluid intake. Fluid spillage during these sessions was corrected for using control bottles located on empty cages.

### Alcohol Consumption

Animals were evaluated for voluntary alcohol consumption over the course of 5 weeks as previously described (van Rijn and Whistler, 2009) and throughout the testing weeks, mice were individually housed in double-grommet ventilated Plexiglas cages for individual fluid consumption. Moderate alcohol intake was tested in the first 3 weeks followed by binge-like consumption in the final 2 weeks. During the first 3 weeks of alcohol testing, animals were offered a two-bottle, limited-access choice between 10% ethyl alcohol and reverse osmosis water for 4 h a day, 2 h into the dark/active phase, where the location of each bottle was alternated daily (right vs. left grommet) to prevent habit formation. Bottles were removed and weighed prior to and after each drinking session; these measurements were recorded Monday–Friday, but only values from Tuesday to Friday were used in the analysis as drinking varied on Mondays following 2 days of forced abstinence. Total daily and weekly average alcohol consumption was recorded for analysis for sex, genotype, and genotype + sex effect.

To measure binge-like-alcohol intake, we used a behavioral model described by Rhodes et al. (2005) where in weeks 4–5 the animals had access to a single bottle of 20% ethyl alcohol for 2-h a day, Monday–Thursday. On Fridays, the duration of the drinking session increased to 4 h. As mentioned for previous fluid consumption experiments, the location of the single 20% alcohol bottle was alternated daily (right vs. left grommet) to prevent habit formation. In these sessions, total alcohol consumed during the 2 weeks of 20% alcohol access was analyzed for sex, genotype, and genotype + sex effect.

### Loss of Righting Reflex

Alcohol naïve animals were moved to a testing room during the dark/active phase and allowed 1 h for acclimation prior to injection with 3.8 g/kg 20% ethyl alcohol (i.p.). For analysis of the sedative hypnotic effects of acute alcohol intoxication, LORR was defined as the inability of a mouse to right itself after being placed on its back three times within 30 s (Ponomarev and Crabbe, 2002). Latency to LORR (time from injection to immobile) and duration of LORR (time from immobile to regaining LORR) were recorded for sex, genotype, and genotype + sex effect.

### Statistical Analysis

All data are presented as means ± standard error of the mean (SEM) using GraphPad Prism 7 software (GraphPad Software, La Jolla, CA, United States). Statistical analysis for the effect of genotype, sex, and sex + genotype interaction was analyzed by two-way ANOVA with either Sidek (genotype differences between the sexes) or Tukey (for within sex genotype differences) post-tests for multiple comparisons.

## Results

### Western Blot Confirmation of β-Arrestin 1 Expression in Different Genotypes and Lower Body Weight in β-Arrestin 1 Knockout Mice

**FIGURE 1:**
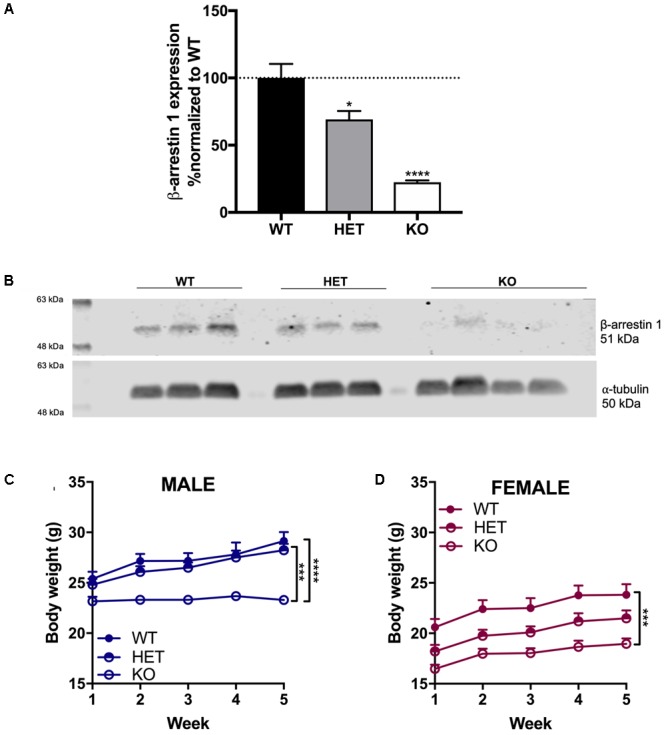
Decreased β-arrestin 1 expression in HET mice and negligible β-arrestin 1 expression in KO mice. Compared to WT, decreased β-arrestin 1 expression was observed via Western blot of cerebellar protein extracts in HET and KO β-arrestin 1 mice **(A)**, as normalized by α-tubulin expression and adjusted for background **(B)**. A decrease in body weight was observed in β-arrestin 1 KO male **(C)** and female **(D)** mice. Significance by one-way ANOVA (β-arrestin 1 Western blot) or two-way ANOVA (body weight over time) with multiple comparisons (Tukey), ^∗^*p* < 0.05, ^∗∗^*p* < 0.01, ^∗∗∗^*p* < 0.001, ^∗∗∗∗^*p* < 0.0001; data represented as mean ± SEM.

To confirm that the HET and homozygous β-arrestin 1 KO mice indeed exhibited altered β-arrestin 1 expression, we measured β-arrestin 1 protein levels from cerebellum in these animals (as high β-arrestin 1 mRNA levels had been measured in male C57Bl/6 previously, Experiment No. 70305395, Allen Institute for Brain Science, 2018) and compared with β-arrestin 1 levels in WT β-arrestin 1 mice, which we normalized as a control (**Figures 1A,B**). We observed a significant decrease in both HET and KO mice β-arrestin 1 expression as determined by one-way, multiple comparisons ANOVA (*F*_2,7_ = 32, *p* = 0.0002, WT vs. HET: *p* = 0.037, WT vs. KO: *p* = 0.0001). Negligible β-arrestin 1 expression observed in KO animals, observed staining at this protein weight is likely non-selective antibody staining of β-arrestin 2, which is also 51 kDa.

Age-matched β-arrestin 1 KO mice of both sexes exhibited decreased body weight compared with WT mice over the course of 5 weeks (**Figures 1C,D**). In male mice, a significant effect of genotype (*F*_2,19_ = 20.8, *p* < 0.0001), time (*F*_4,76_ = 25.2, *p* < 0.0001), and interaction (*F*_8,76_ = 6.39, *p* < 0.0001) was found, where multiple comparisons revealed a significant difference between KO vs. WT (*p* < 0.0001) and vs. HET (*p* = 0.0002). In females, a similar decrease in body weight was observed in β-arrestin 1 KO mice with a significant effect of genotype (*F*_2,21_ = 12.2, *p* = 0.0003) and time (*F*_4,84_ = 25.2, *p* < 0.0001). No interaction (*F*_8,84_ = 1.04, *p* < 0.0001) effect was observed, although multiple comparisons revealed a significant difference in body weight between KO vs. WT (*p* = 0.0002).

### Increased Locomotor Activity in β-Arrestin 1 Knockout Animals With No Significant Differences in Open-Field Anxiety Behavior Compared With Wild-Type or Heterozygous

**FIGURE 2:**
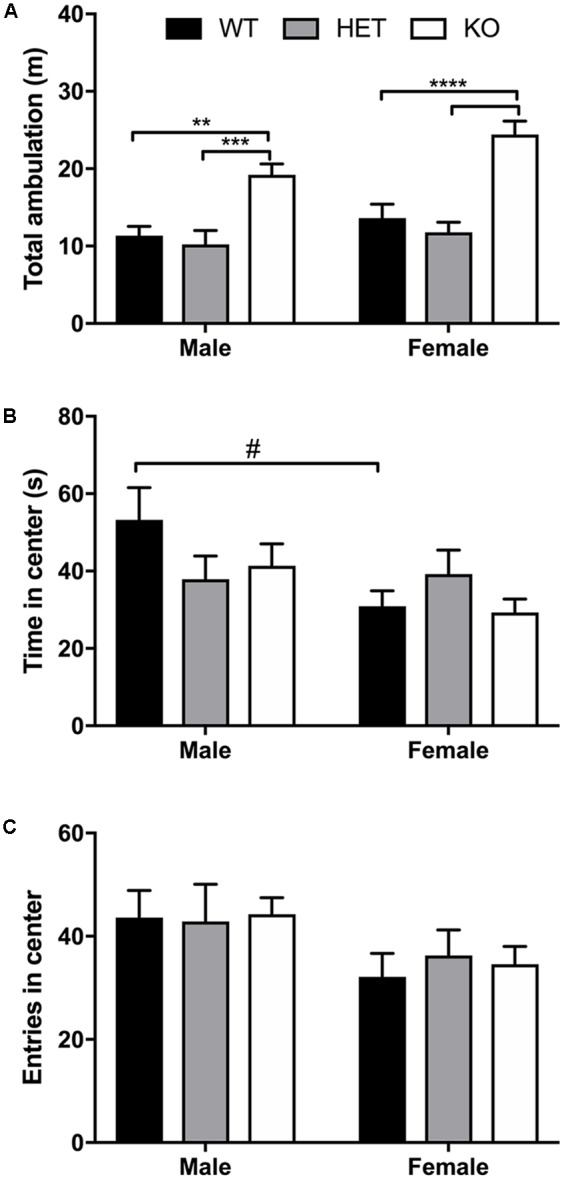
Increased locomotor activity in β-arrestin 1 KO animals but no genotype effect on anxiety-like behavior. Locomotor activity was assessed for WT, HET, and β-arrestin 1 KO male and female C57BL/6 mice in a 60-min session. An overall sex effect in total locomotion were observed (*n* = 27 males, *n* = 25 females), and KO male mice (*n* = 10) displaying higher ambulation than WT (*n* = 8) or HET (*n* = 9) males **(A)**. This genotype effect was also observed between female KO mice (*n* = 9) and WT (*n* = 8) and HET (*n* = 7) females **(A)**. Male mice spent more time in the center of the arena than females during the first 5 min of testing **(B)**, although no genotype effect was observed. Similarly, males entered the center area of the arena more times than females, although again no genotype effect was noted **(C)**. Significance by two-way ANOVA with multiple comparisons (Tukey within sex, Sidek between genotype), ^∗∗^*p* < 0.01, ^∗∗∗^*p* < 0.001, ^∗∗∗∗^*p* < 0.0001; ^#^*p* < 0.05, data represented as mean ± SEM.

For the 60-min locomotor session, a significant effect of genotype (*F*_2,46_ = 29.4, *p* < 0.0001) and sex (*F*_1,46_ = 5.54, *p* = 0.0229) was found, with no significant interaction effect (*F*_2,46_ = 0.810, *p* = 0.451) (**Figure 2A**). Within males, a significant increase in locomotion was observed between KO and both WT (*p* = 0.0022) and HET (*p* = 0.0003) with no significant difference in total ambulation between WT and HET (*p* = 0.866). In females, KO mice ambulated significant more than WT (*p* < 0.0001) and HET (*p* < 0.0001) with no significant difference in ambulation between WT and HET (*p* = 0.703). For time spent in the center of the open-field box in the first 5 min, a significant effect of sex (*F*_1,46_ = 5.39, *p* = 0.0248) was observed as male spent more time in the center than females, although no significant effect of genotype (*F*_2,46_ = 0.706, *p* = 0.499) or interaction (*F*_2,46_ = 1.96, *p* = 0.153) was noted (**Figure 2B**). Within genotypes, male WT mice ambulated significantly more than female WT mice (*p* = 0.033). For number of entries into the center quadrant during the first 5 min, a similar sex effect was found (*F*_1,46_ = 5.36, *p* = 0.0251) as males entered the center more than females, but no effect of genotype (*F*_2,46_ = 0.070, *p* = 0.932) or interaction (*F*_2,46_ = 0.121, *p* = 0.886) (**Figure 2C**) was observed.

### β-Arrestin 1 Male Knockout Animals Spend More Time in Light With Higher Ambulation in Light/Dark Transition Test

**FIGURE 3:**
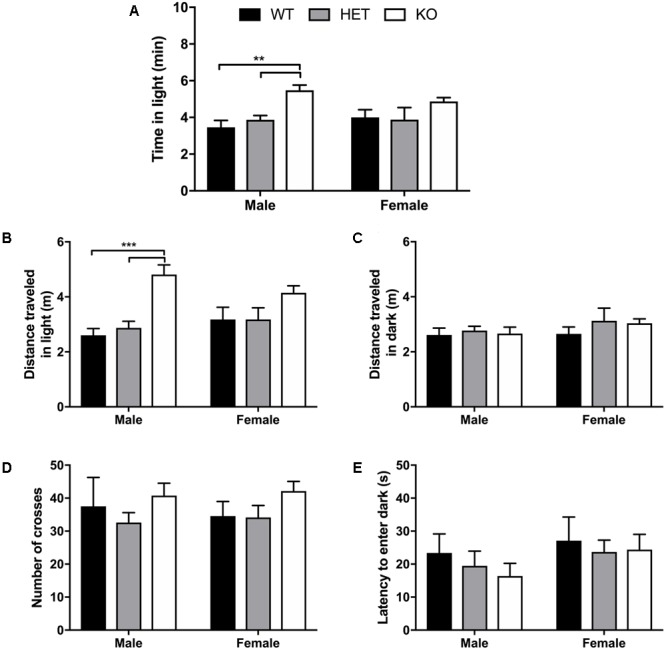
Light/dark transition test for anxiety-like behavior reveals male β-arrestin 1 KO animals spend more time in anxiogenic compartment with higher locomotor. Light/dark transition was performed for WT, HET, and β-arrestin 1 KO male and female C57BL/6 mice in a 10-min session. No sex effect was observed for time spent in the light (anxiogenic) compartment (*n* = 26 males, *n* = 29 females) but a significant genotype effect was observed between KO male mice (*n* = 10) and WT (*n* = 6) and HET male mice (*n* = 10) **(A)**. No genotype effect was observed between female β-arrestin 1 WT (*n* = 7), HET (*n* = 7), or KO mice (*n* = 15) **(A)**. Total ambulation in the light (anxiogenic) compartment was significantly increased in β-arrestin 1 KO males compared with male WT or HET mice, with no genotype effect observed in females **(B)**. No sex or genotype effects were observed for distance traveled in the dark (anxiolytic) compartment **(C)**. No sex or genotype differences were observed for number of crosses between the light and dark compartments **(D)** or latency to enter the dark (anxiolytic) compartment **(E)**. Significance by two-way ANOVA with multiple comparisons (Tukey within sex, Sidek between genotype), ^∗∗^*p* < 0.01, *^∗∗∗^p* < 0.001; data represented as mean ± SEM.

No sex (*F*_1,49_ = 0.00470, *p* = 0.946) nor interaction of sex + genotype effect (*F*_2,49_ = 1.37, *p* = 0.264) was detected for time spent in the light compartment during the 10 min light/dark transition test, although a genotype effect (*F*_2,49_ = 11.7, *p* < 0.0001) was observed (**Figure 3A**). Multiple comparisons test revealed that male β-arrestin 1 KO spent more time in the light (anxiogenic) compartment compared with male WT (*p* = 0.0011) or male HET (*p* = 0.0028) mice. For total distance traveled in the light compartment, a significant effect of genotype (*F*_2,49_ = 15.5, *p* < 0.0001) was observed with no effect of sex (*F*_1,49_ = 0.0632, *p* = 0.803) nor interaction (*F*_2,49_ = 2.06, *p* = 0.138) (**Figure 3B**). Multiple comparisons revealed that this genotype effect was prevalent between male β-arrestin 1 KO and WT (*p* = 0.0002) and HET (*p* = 0.0002), with higher ambulation in the light in male KO mice. This significant effect of genotype was not observed in distance traveled in the dark compartment, as no effect of genotype (*F*_2,49_ = 0.709, *p* = 0.497), sex (*F*_1,49_ = 01.54, *p* = 0.221), or interaction (*F*_2,49_ = 0.249, *p* = 0.781) was observed (**Figure 3C**). For number of crosses between the two compartments, no sex (*F*_1,49_ = 3.87 × 10^-6^, *p* = 0.998), genotype (*F*_2,49_ = 2.18, *p* = 0.125), or interaction effect (*F*_2,49_ = 0.154, *p* = 0.857) was noted (**Figure 3D**). A similar lack of sex (*F*_2,49_ = 1.53, *p* = 0.223), genotype (*F*_1,49_ = 0.425, *p* = 0.656), or interaction effect (*F*_2,49_ = 0.116, *p* = 0.891) was observed for latency to enter the dark compartment (**Figure 3E**). Overall, these results suggest that the increased locomotor activity observed in male β-arrestin 1 KO accounted for the increase in time spent in the light rather than a difference in anxiety-like behaviors.

### No Differences in Sucrose Preference Between β-Arrestin 1 Genotypes

**FIGURE 4:**
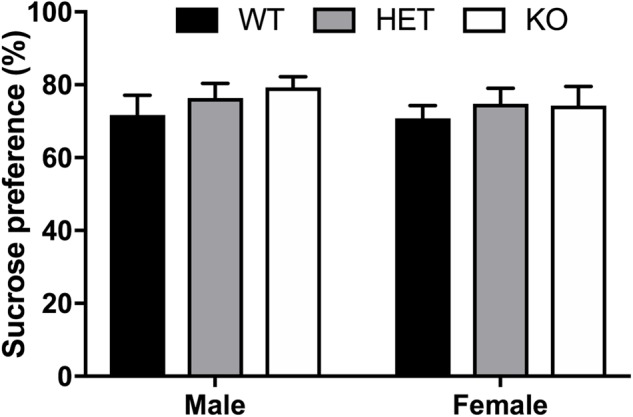
No sex or β-arrestin 1 genotype effect on preference of natural reward, sucrose. A two-bottle, limited access procedure of increasing concentrations of sucrose (0.25–4%) for a total of 10 days revealed no sex (*n* = 26 males, *n* = 24 females), genotype, or interaction effect on average sucrose preference. Significance by two-way ANOVA with multiple comparisons (Tukey within sex, Sidek between genotype); data represented as mean ± SEM.

No effect of sex (*F*_1,53_ = 0.501, *p* = 0.482), genotype (*F*_2,53_ = 0.2943, *p* = 0.396), or interaction (*F*_2,53_ = 0.140, *p* = 0.870) was observed for total sucrose consumption within β-arrestin 1 (**Figure 4**), suggesting no alterations in natural reward intake behavior. To assess if differences in general fluid intake were present, we measured total water intake during the 20-h access period to water alone. No significant difference in 20-h water intake was noted by volume alone (sex: *F*_1,53_ = 0.0194, *p* = 0.890; genotype: *F*_2,53_ = 0.*157, p* = 0.855; interaction: *F*_2,53_ = 2.52, *p* = 0.0897, Supplementary Figure 1A) or by volume consumed per body weight for genotype (*F*_2,53_ = 2.00, *p* = 0.146) or interaction (*F*_2,53_ = 1.52, *p* = 0.227), although female mice consumed more water per body weight than males (sex: *F*_1,53_ = 16.4, *p* = 0.0002) (Supplementary Figure 1B).

### Ethanol Consumption Is Higher in Female Animals in General and β-Arrestin 1 Expression Prevents Increased Binge Consumption in Females

**FIGURE 5:**
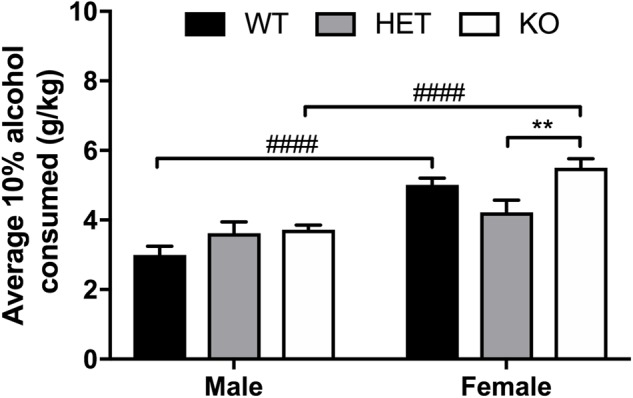
Average daily 10% alcohol intake in a limited access model reveals large overall sex effect, with β-arrestin 1 KO females consuming more alcohol than HET females. Animals were trained to consume 10% alcohol in a two-bottle, limited access (4 h a day, 5 days a week), drinking-in-the-dark model for 3 weeks. Average daily alcohol consumption was significantly higher in female (*n* = 29) mice compared with male mice (*n* = 28). No overall genotype effect was observed between male animals (*n* = 7 WT, *n* = 10 HET, *n* = 11 KO) although KO female mice consumed significantly more alcohol than HET females (*n* = 9 WT, *n* = 10 HET, *n* = 11 KO). When comparing the two sexes within the same genotype, female WT or KO consumed more alcohol that male WT or KO, respectively. Significance by two-way ANOVA with multiple comparisons (Tukey within sex, Sidek between genotype), ^∗∗^*p* < 0.01, ^####^*p* < 0.0001; data represented as mean ± SEM.

**FIGURE 6:**
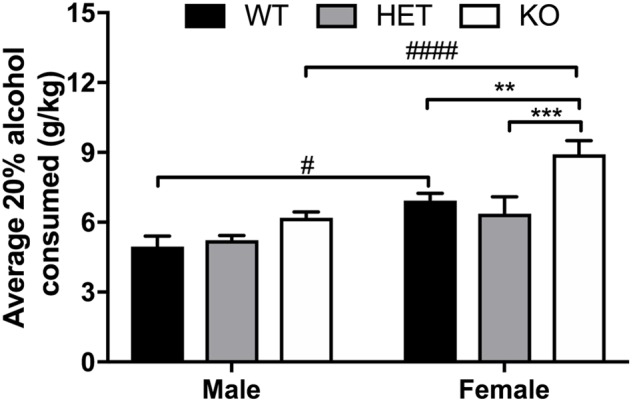
Overall sex effect on binge 20% intake and binge 20% alcohol intake is higher in female β-arrestin 1 KO animals. Following limited access training to 10% alcohol, animals were exposed to 20% alcohol in a single-bottle, binge access (2 h a day, 4 days a week – 4 h a day, 1 day a week), drinking-in-the-dark model for 2 weeks. Average binge intake during the 4-h session was significantly higher in females (*n* = 27) compared with male mice (*n* = 25) as observed by an overall sex effect. In males, KO males (*n* = 11) did not drink significantly more than WT (*n* = 7) or HET (*n* = 7). Female KO mice (*n* = 11) consumed more alcohol than WT (*n* = 9) and HET (*n* = 7) females. When comparing the two sexes within the same genotype, female WT or KO mice also consumed more alcohol than male WT or KO mice, respectively. Significance by two-way ANOVA with multiple comparisons (Tukey within sex, Sidek between genotype), ^∗∗^*p* < 0.01, ^∗∗∗^*p* < 0.001; ^#^*p* < 0.05, ^####^*p* < 0.00001; data represented as mean ± SEM.

An overall sex effect was observed between male and female animals for average alcohol intake in the limited access, 10% two-bottle choice protocol (*F*_1,51_ = 46.45, *p* < 0.0001), as well as a genotype (*F*_2,51_ = 4.52, *p* = 0.0157) and interaction effect (*F*_2,51_ = 4.11, *p* = 0.0221). Within male animals, no observed differences in average alcohol intake were present, yet HET female mice consumed less 10% alcohol (*p* = 0.0025) on average than KO female animals (**Figure 5**). Across genotypes, female KO mice consumed more alcohol than male KO (*p* < 0.0001). Female WT mice also consumed more alcohol than male WT (*p* < 0.0001). In the binge 20% alcohol consumption model, a significant sex effect was again observed (*F*_1,46_ = 25.9, *p* < 0.0001). An overall genotype effect (*F*_2,46_ = 9,83, *p* < 0.0003) was observed although no interaction effect was noted (*F*_2,46_ = 1.50, *p* < 0.233). Within male animals, no significant genotype effect was observed by multiple comparisons, although within females, β-arrestin 1 KO mice consumed more alcohol than WT (*p* = 0.0056) and HET (*p* = 0.0009) (**Figure 6**). Furthermore, within genotypes, a significant increase in alcohol intake was observed between female β-arrestin 1 KO compared with male β-arrestin 1 KO (*p* < 0.0001) and female WT compared to male WT (*p* = 0.0168).

### Decreased Latency to Loss of Righting Reflex in β-Arrestin 1 Knockout Mice But No Alterations in Duration of LORR

**FIGURE 7:**
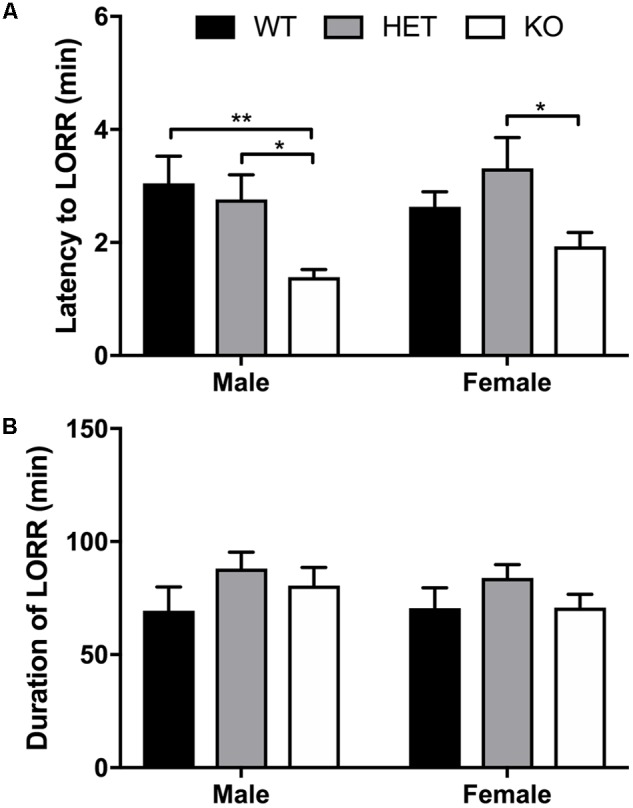
Decreased latency to LORR observed in β-arrestin 1 KO mice but duration of LORR not affected by β-arrestin 1 expression. Following 3.8 g/kg 20% ethanol administration (i.p.), no sex effect was observed for latency to LORR (*n* = 26 males, *n* = 25 females), although a genotype effect was observed in both male (*n* = 7 WT, *n* = 8 HET, *n* = 10 KO) and female mice (*n* = 8 WT, *n* = 8 HET, *n* = 8 KO), with decreased latency was observed in mice lacking β-arrestin 1 expression **(A)**. Duration of LORR was not affected by sex or β-arrestin 1 genotype alone, nor were differences observed by genotype + sex **(B)**. Significance by two-way ANOVA with multiple comparisons (Tukey within sex, Sidek between genotype), ^∗^*p* < 0.05, ^∗∗^*p* < 0.01; data represented as mean ± SEM.

No sex (*F*_1,44_ = 0.596, *p* = 0.448) or interaction (*F*_2,44_ = 1.10, *p* = 0.342) effect was observed for latency to LORR, although a significant genotype effect (*F*_2,42_ = 9,07, *p* = 0.0005) was noted (**Figure 7A**). Multiple comparisons revealed that male β-arrestin 1 KO mice exhibited decreased latency to LORR compared with male WT (*p* = 0.0008) and HET (*p* = 0.0177). In female mice, multiple comparisons revealed that female β-arrestin 1 KO mice had decreased latency to LORR compared with female HET (*p* = 0.0249) but not WT (*p* = 0.367). For duration of LORR, no sex (*F*_1,44_ = 0.439, *p* = 0.511), genotype (*F*_2,44_ = 2.08, *p* = 0.137), or interaction (*F*_2,44_ = 0.238, *p* = 0.790) effect was observed (**Figure 7B**).

## Discussion

Using a global KO strategy, we explored the role of β-arrestin 1 in relation to basal anxiety-like behavior and behavior associated with consumption of rewarding substances. A potential pitfall when utilizing a global, congenic KO strategy is that mice may display altered cellular or behavioral responses resulting from compensatory adaptations to cope with the absence of β-arrestin 1 expression. However, currently no conditional β-arrestin 1 isoform KOs have been produced to overcome this.

Here, we observed increased baseline locomotion in both male and female β-arrestin 1 KO mice and decreased trait anxiety-like behavior in male β-arrestin 1 KO mice. In our animals, we observed that male mice without β-arrestin 1 expression exhibit decreased trait anxiety, as measured by increased time spent in the more anxiogenic light compartment in the light/dark test (**Figure 3**), although this increased time spent in the light compartment may be the result of increased locomotor activity in general (**Figures 2, 3**). However, one study has reported that anxious mice display reductions in β-arrestin 1 protein levels in blood plasma (Mendez-David et al., 2013). For alcohol consumption, a large sex effect was observed with female animals consuming more alcohol than male animals in both limited access and binge alcohol models, as observed by other studies (Middaugh and Kelley, 1999; Yoneyama et al., 2008; Gelineau et al., 2017). A recent study by Mittal et al. (2017) observed that β-arrestin 1 expression is essential for regulating reward-motivated behaviors associated with cocaine self-administration and natural food reward through a mechanism associated with altered glutamatergic function, as β-arrestin 1 KO mice exhibited deficits in both types of reward responding. This observed deficit in natural reward intake may be reflected in the decreased body weights observed in our β-arrestin 1 KO mice compared with WT (**Figure 1**). However, we did not observe differences for sucrose preference (**Figure 4**), in contrast with the previously observed decrease in natural food reward in these mice (Mittal et al., 2017). The discrepancies in the observed behavior of β-arrestin 1 KO mice may be the result of differences related to the type of learning involved in operant self-administration (Pavlovian) versus volitional intake assays (non-Pavlovian). Surprisingly, in our voluntary alcohol consumption assays, we did observe that female KO β-arrestin 1 mice consumed more alcohol (**Figures 5, 6**) than WT (for binge) or HET (for limited access and binge) females. These results are suggestive of a potential protective role of β-arrestin 1 expression in increased voluntary alcohol intake or protective genetic compensation upon β-arrestin 1 KO in females. These alterations in alcohol consumption are not believed to be the result of differences in alcohol metabolism, as no differences in the time it takes for animals to regain consciousness following alcohol sedation in a loss of duration of righting reflex assay were apparent by genotype or sex, although β-arrestin 1 KO mice were quicker to sedate to alcohol compared with WT (in male mice only) and HET (in both sexes), which may indicate increased GABAa receptor function (Davies and Alkana, 2001) in the KO animals.

We can speculate on what is driving the ability of β-arrestin 1 to modulate certain behaviors; β-arrestin 1 has been implicated in signaling events requiring translocation to the nucleus, such as increased Bcl2, P27, arachidonic acid, and ROCK/LIMK signaling (Kang et al., 2005; Shi et al., 2007; Mittal et al., 2013).

The effects of β-arrestin 1 expression in neuronal cell survival following a variety of neurological insults remain to be more fully investigated, although increased β-arrestin 1 expression has been correlated with decreased negative neurological behavioral effects following ischemic insult (Wang et al., 2014). β-Arrestin isoform selective signaling has been previously observed at the metabotropic glutamate receptor 7 (mGlu7) *in vitro*, where β-arrestin 1 signaling increased ERK1/2 and inhibited JNK, while β-arrestin 2 performed the opposite (Iacovelli et al., 2014), although this reciprocal regulation may be cell-type and receptor specific (Kohout et al., 2001), as *in vivo* β-arrestin 2 disruption decreased murine hippocampal metabotropic glutamate receptor 5 (mGlu5)-associated ERK activation (Stoppel et al., 2017).

Besides differences in receptor-mediated downstream signaling, varied responses to drug exposure and disease have been observed between β-arrestin 1 and 2 using *in vivo* and *in vitro* models. For example, amphetamine-induced hyperlocomotion is initially enhanced in male β-arrestin 1 KO mice while β-arrestin 2 KO mice are hyposensitive to amphetamine and display poor locomotor sensitization (Beaulieu et al., 2005; Zurkovsky et al., 2017). Also, expression of β-arrestin 2, but not β-arrestin 1, is required for morphine-induced hyperlocomotion (Urs et al., 2011), where interestingly, expression of β-arrestin 2 increases morphine, but not cocaine, reward (Bohn et al., 2003). Our behavioral results suggest that selectively activating β-arrestin 1 recruitment and/or signaling may result in decreased alcohol intake in females and prevent locomotor hyperactivity. We have previously observed that β-arrestin 2 recruitment at the δ-opioid receptor is associated with increased alcohol intake (Chiang et al., 2016). Similarly, β-arrestin 2 KO mice have been reported to consume less alcohol and mice bred to prefer alcohol display increased β-arrestin 2 expression (Bjork et al., 2008). In the opioid field, there has been a push to develop G-protein biased drugs that do not recruit β-arrestin (Soergel et al., 2013, 2014; Manglik et al., 2016; Singla et al., 2016); however, our data suggest that sometimes it may be beneficial to selectively recruit β-arrestin 1, i.e., selectively avoid β-arrestin 2 recruitment. A few recent studies suggest that it may be possible to identify drugs that preferentially recruit a specific β-arrestin isoform upon receptor binding. Agonist-selective recruitment of β-arrestin isoforms has been observed at the δ-opioid receptor, where high-internalizing agonists recruit β-arrestin 1 to the receptor while low-internalizing agonists preferentially recruit β-arrestin 2 (Pradhan et al., 2016). Additionally, reports of the δ-opioid agonist etorphine suggest that this alkaloid agonist – compared with peptide agonists such as [D-Pen2,D-Pen5]enkephalin (DPDPE) and deltorphin I – promotes δ-opioid receptor endocytosis in a β-arrestin 1-dependent manner which is not observed upon DPDPE or deltorphin I activation, although β-arrestin 2 was not expressed in this study and therefore β-arrestin 2-dependent effects were not discussed (Aguila et al., 2012).

In summary, here we provide additional support that β-arrestin 1 may have similar, as well as unique, roles in behavior compared with β-arrestin 2, as described in β-arrestin 2 studies previously conducted. Our study also revealed instances of β-arrestin 1-associated sex differences, such as those described here for alcohol intake, suggesting that it is important to consider both sex and β-arrestin isoforms when studying biased signaling at GPCRs. Our studies warrant continued investigation into the mechanisms that underlie these observed behavioral differences in β-arrestin 1 KO mice and potential underlying differences in downstream signaling cascades between the two β-arrestin isoforms. There is an increased interest in developing β-arrestin-biased agonists for specific indications (Shukla et al., 2008; Allen et al., 2011; Abraham et al., 2016; Liu et al., 2017; Mannel et al., 2017a); ours and future studies could promote the idea of developing of β-arrestin-isoform-biased agonists.

## Author Contributions

MR designed the experiments, performed Western blot and behavioral experiments, analyzed and interpreted the data, and wrote the main draft of the manuscript. TC conducted the behavioral experiments, analyzed and interpreted the data, and bred the mice. MK and JB bred the mice. JH analyzed and interpreted the data. RvR designed the experiments, analyzed and interpreted the data, and edited the manuscript.

## Conflict of Interest Statement

The authors declare that the research was conducted in the absence of any commercial or financial relationships that could be construed as a potential conflict of interest.

## Abbreviations

CPP: conditioned place preference
GPCR: G protein-coupled receptor
HET: heterozygous
KO: knockout
LORR: loss of righting reflex
WT: wild-type
