# Supplementary material for: Behavioral Characterization of β-Arrestin 1 Knockout Mice in Anxiety-Like and Alcohol Behaviors
Source: Front Behav Neurosci. 2018 Mar 20;12:54. doi: 10.3389/fnbeh.2018.00054 (PMC5869203; doi:10.3389/fnbeh.2018.00054)
Supplement: Supplementary file 1 [file Image_1.PDF]

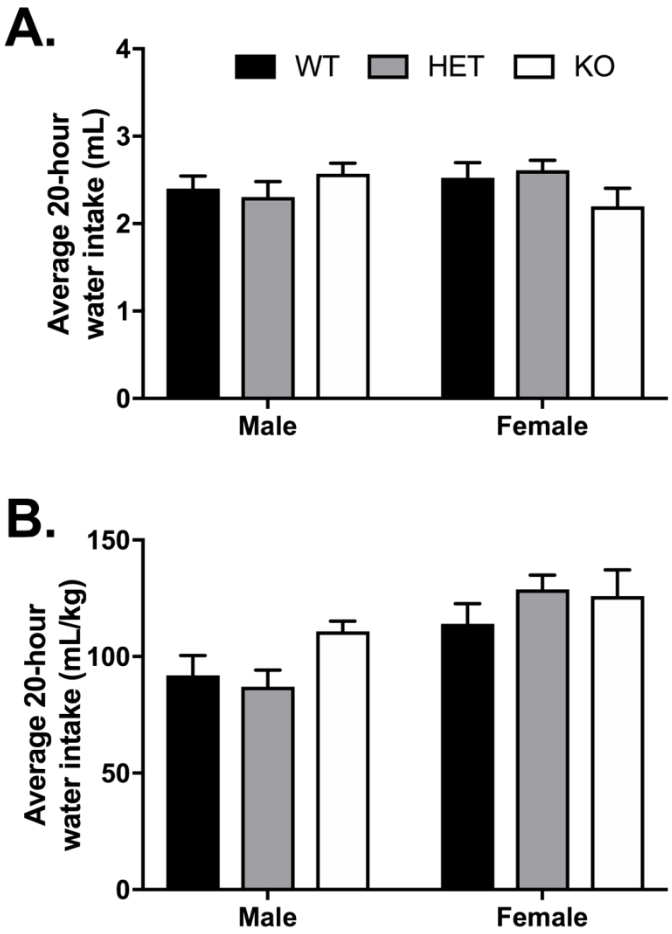

Supplemental Figure 1. No differences in average water intake by volume or average water intake by volume/body weight. Average 20-hour water intake by volume (**A**) or by volume/body weight (**B**) was not significantly different between β-arrestin 1 genotypes in male or female mice. Significance by two-way ANOVA with multiple comparions (Tukey within sex, Sidek between genotype); data represented as mean ± SEM.
